# Cost yield of different treatment strategies against *Clonorchis sinensis* infection

**DOI:** 10.1186/s40249-021-00917-1

**Published:** 2021-12-22

**Authors:** Men-Bao Qian, Chang-Hai Zhou, Hui-Hui Zhu, Ying-Dan Chen, Xiao-Nong Zhou

**Affiliations:** 1 National Institute of Parasitic Diseases, Chinese Center for Disease Control and Prevention, Shanghai, China; 2 Chinese Center for Tropical Diseases Research, Shanghai, China; 3 Key Laboratory of Parasite and Vector Biology, National Health Commission, Shanghai, China; 4 National Center for International Research on Tropical Diseases, Ministry of Science and Technology, Shanghai, China; 5 WHO Collaborating Center for Tropical Diseases, Shanghai, China; 6 School of Global Health, Chinese Center for Tropical Diseases Research, Shanghai Jiao Tong University School of Medicine, Shanghai, China

**Keywords:** *Clonorchis sinensis*, Treatment strategy, Cost effectiveness, Cost utility, Disability-adjusted life years

## Abstract

**Background:**

Clonorchiasis is attributed to the ingestion of raw freshwater fish harboring *Clonorchis sinensis*. Morbidity control is targeted through the administration of antihelminthics. This study modelled the cost yield indicated by effectiveness and utility of different treatment strategies against clonorchiasis.

**Methods:**

About 1000 participants were enrolled from each of 14 counties selected from four provincial-level administrative divisions namely Guangxi, Guangdong, Heilongjiang and Jilin in 2017. Fecal examination was adopted to detect *C. sinensis* infection, while behavior of ingesting raw freshwater fish was enquired. Counties were grouped into four categories based on prevalence, namely low prevalence group (< 1%), moderate prevalence group (1–9.9%), high prevalence group (10–19.9%) and very high prevalence group (≥ 20%), while population were divided into three subgroups, namely children aged below 14 years old, adult female and adult male both aged over 14 years old. The average of cost effectiveness indicated by the cost to treat single infected cases with *C. sinensis* and of cost utility indicated by the cost to avoid per disability-adjusted life years (DALYs) caused by *C. sinensis* infection was calculated. Comparisons were performed between three treatment schedules, namely individual treatment, massive and selective chemotherapy, in which different endemic levels and populations were considered.

**Results:**

In selective chemotherapy strategy, the cost to treat single infected case in very high prevalence group was USD 10.6 in adult male, USD 11.6 in adult female, and USD 13.2 in children. The cost increased followed the decrease of endemic level. In massive chemotherapy strategy, the cost per infected case in very high prevalence group was USD 14.0 in adult male, USD 17.1 in adult female, USD 45.8 in children, which were also increased when the endemic level decreased. In individual treatment strategy, the cost was USD 12.2 in adult male, USD 15.0 in adult female and USD 41.5 in children in very high prevalence group; USD 19.2 in adult male, USD 34.0 in adult female, and USD 90.1 in children in high prevalence group; USD 30.4 in adult male, USD 50.5 in adult female and over USD 100 in children in moderate prevalence group; and over USD 400 in any population in low prevalence group. As to cost utility, the differences by treatment strategies, populations and endemic levels were similar to those in cost effectiveness.

**Conclusions:**

Both cost effectiveness and cost utility indicators are highly impacted by the prevalence and population, as well as the treatment schedules. Adults especially men in the areas with a prevalence over 10% should be prioritized, in which selective chemotherapy was best and massive chemotherapy was also cost effective. In moderate endemic areas, the yield is not ideal, but selective chemotherapy for adult male may also be adopted. In low endemic areas, all strategies were high costly and new strategies need to be developed.

**Graphical Abstract:**

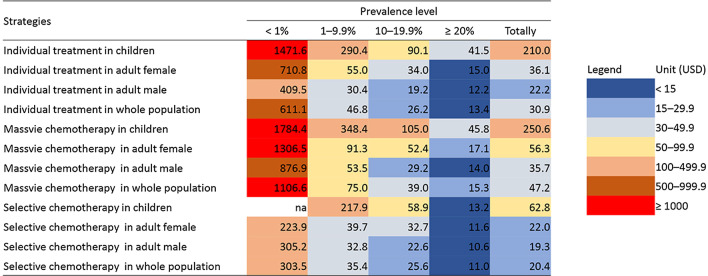

**Supplementary Information:**

The online version contains supplementary material available at 10.1186/s40249-021-00917-1.

## Background

Infections with human liver fluke (*Clonorchis sinensis*, *Opisthorchis viverrini* and *O. felineus*) cause high burden in Asia and parts of Europe [1–3]. They are caused by the special dietary habit-ingesting raw or undercooked freshwater fish. Especially, an estimation of 15 million people is infected with *C. sinensis* across China, Republic of Korea, northern Vietnam and part of Russia [4–6]. Diverse morbidities are associated with *C. sinensis* infection, among which gallstone, cholecystitis, cholangitis, and cholangiocarcinoma are most important [7–10]. An average loss of 7.5% in health could be attributable to *C. sinensis* infection [11].

High burden due to severe morbidity and availability of antihelminthics lead to the target of morbidity control through chemotherapy [12–14]. Preventive chemotherapy effectively decreases the infection and intensity. A dosage of 75 mg/kg praziquantel divided into three doses in 1 day is usually applied for both individual and population treatment [13, 15, 16]. Two different strategies could be chosen in preventive chemotherapy, namely mass chemotherapy for whole communities and selective one for people at risk in the communities [13]. Usually, persons ingesting raw freshwater fish frequently are considered at risk [17]. Preventive chemotherapy is not based on the individual definitive diagnosis, and thus it is usually applied when the prevalence reaches a threshold. Compared to preventive chemotherapy, individual treatment is used when infection is ascertained through definite diagnosis, i.e. detection of eggs in feces [15, 16].

By now, only a few studies have been implemented to compare the cost effectiveness of different treatment schedules (individual treatment, massive and selective chemotherapy) against human liver fluke infections [18]. No study has yet considered the impact from different populations (gender and ages). Especially, no cost utility analysis based on disability-adjusted life years (DALYs) has yet been implemented. In a previous study, we had demonstrated the quantitative contribution of ingesting raw freshwater fish to *C. sinensis* infection and the screening performance of detecting *C. sinensis* cases through raw-freshwater fish-eating practice [17, 19]. Here, the data were used to compare the cost effectiveness and cost utility of three different treatment schedules, in which the impact from prevalence levels and populations was also considered.

## Methods

### Study areas and participants

The study areas had been described elsewhere [17, 19]. In brief, four major clonorchiasis endemic provincial-level administrative divisions (PLADs) in China, namely Guangxi and Guangdong in southeastern regions and Heilongjiang and Jilin in northeastern areas, were selected. Correspondingly, 6, 3, 5 and 3 counties were selected from each PLAD. In each county, five village were selected and then about 200 villagers from each village were included in the survey.

### Investigation procedures

In 2017, each participant was asked to provided one fresh feces, which was then transferred to local medical organization and examined by technicians using the Kato-Katz method with a template of 41.7 mg [20, 21]. Two smears were prepared for each sample. Each participant was also inquired of the habit of ingesting raw freshwater fish.

The cost was based on the average unit on each item which had been applied in filed. The cost contained the expenditure on fecal examination, behavioral screening, purchase and delivery of drugs (praziquantel). The cost on fecal examination per person (including the labor expenditure and material expenditure) was CNY 20 (USD 3.10), while the cost on behavioral screening was CNY 1 (USD 0.16). The cost on drugs was CNY 170 per bottle including 100 tablets (200 mg individually), namely USD 0.26 per tablet. The delivery of drugs costed CNY 2 (USD 0.31) individually.

### Statistical analysis

Data were analyzed in SPSS for Windows (version 11.0; SPSS Institute, Inc., Chicago, USA) and Microsoft Excel (version 2016; Microsoft Corporation, Redmond, USA). One county was excluded because people reported ingestion of marine fish and the prevalence of *C. sinensis* was 0. Another two counties were also excluded because no *C. sinensis* infection was detected. Finally, 14 counties were included in this study, and they were classified into four groups based on prevalence, namely low prevalence group (< 1%), moderate prevalence group (1–9.9%), high prevalence group (10–19.9%) and very high prevalence group (≥ 20%) level. Population was divided into three categories, namely children (≤ 14 years old), adult female (> 14 years old) and adult male (> 14 years old) [22]. An average body weight was 35 kg in children, 55 kg in adult female and 65 kg in adult male [23]. A total of 75 mg/kg praziquantel divided into three doses in 1 day was set to be administrated in all three treatment schedules [13, 15, 16].

This study modelled the cost effectiveness and cost utility stratified by treatment schedules, counties, endemic levels, and populations. DALYs was introduced as utility indicator, which includes years of life living with a disability (YLDs) and years of life lost (YLLs) [24].

$$DALYs=YLDs+YLLS$$

$$YLDs=N \times P \times D$$

where *N* stands for community population, *P* the prevalence of *C. sinensis* and *D* the average disability weight of *C. sinensis* infection.

$$YLLs=N \times P \times I \times (L-a)$$

where *N* stands for community population, *P* the prevalence of *C. sinensis*, *I* the incidence of cholangiocarcinoma attributed to *C. sinensis* infection, *L* the standard expectation of the life and *a* the age at death of those with cholangiocarcinoma.

Eggs per gram of feces (EPG) was calculated by multiplying the average egg number of two smears by 24. EPG was logarithmically transformed and the average was calculated for different groups, which was then inversely logarithmically transformed to capture the geometric mean of EPG (GMEPG). Then, the disability weight was captured based on the equation of *D* = 0.0362ln(*GMEPG*) − 0.1269 [11]. Because minus figures occurred when the equation was extrapolated to low GMEPG, thus a lower limit was set as 0.022 [11]. This is reasonable, because the loss of health in low infection intensity was completed due to diarrhea and pain in the right upper quadrant, which are common in those infected with *C. sinensis*. The period of disease was set as 1 year, because the prevalence instead of incidence was used in this study. To calculate the YLLs, death due to *C. sinensis* infection was completely attributed to cholangiocarcinoma, with an incidence of 25/100 000 and 35/100 000 in female and male respectively [25]. Because the progress of cholangiocarcinoma is chronic and thus YLLs was not considered in children. The life expectancy was 79.92 in female and 74.52 in male [26], when the onset age of cholangiocarcinoma was referred to liver cancer namely 62.35 in female and 68.99 in male [27]. Because the prognosis is very poor in cholangiocarcinoma, the death age was set equally to onset age of cholangiocarcinoma. The cost of individual treatment contained fecal examination, purchase and delivery of drugs for those with *C. sinensis* infection, the cost of massive chemotherapy contained purchase and delivery of drugs for whole populations, and that of selective chemotherapy contained behavioral screening and purchase and delivery of drugs for those ingesting raw freshwater fish. Cost to treat individual infected case with *C. sinensis* was used as the indicator in cost effectiveness analysis, while cost to avoid one YLDs, YLLs and DALYs as the indicator in cost utility analysis. The average of cost effectiveness and cost utility was calculated and compared, which was stratified by the three treatment schedules (i.e., individual treatment, massive and selective chemotherapy), endemic levels and populations. The composition of cost was also analyzed, with the cost in each category divided by the overall cost.

## Results

### Epidemiological profiles

The prevalence of *C. sinensis* infection and the proportion of persons ingesting raw freshwater fish refer to Additional file 1: Table S1 [17, 19]. The epidemiological profiles of *C. sinensis* prevalence and raw-freshwater fish-eating practice were similar in different endemic levels, namely higher prevalence of *C. sinensis* and proportion of raw-fish-eating practice in male compared to female and in elder people compared to children.

Overall, the DALYs per 1000 was 6.4, ranging from 0.2 to 34.3 in different counties. It was 0.5 in children ranging from 0 to 7.3, 4.6 in adult female ranging from 0 to 26.0, and 10.5 in adult male ranging from 0.2 to 42.9 (Additional file 1: Table S2). In adult female, the YLDs per 1000 was 4.3 and the YLLs per 1000 was 0.3. In adult male, the YLDs per 1000 was 9.7 and the YLLs per 1000 was 0.8.

### Cost effectiveness

**Fig. 1:**
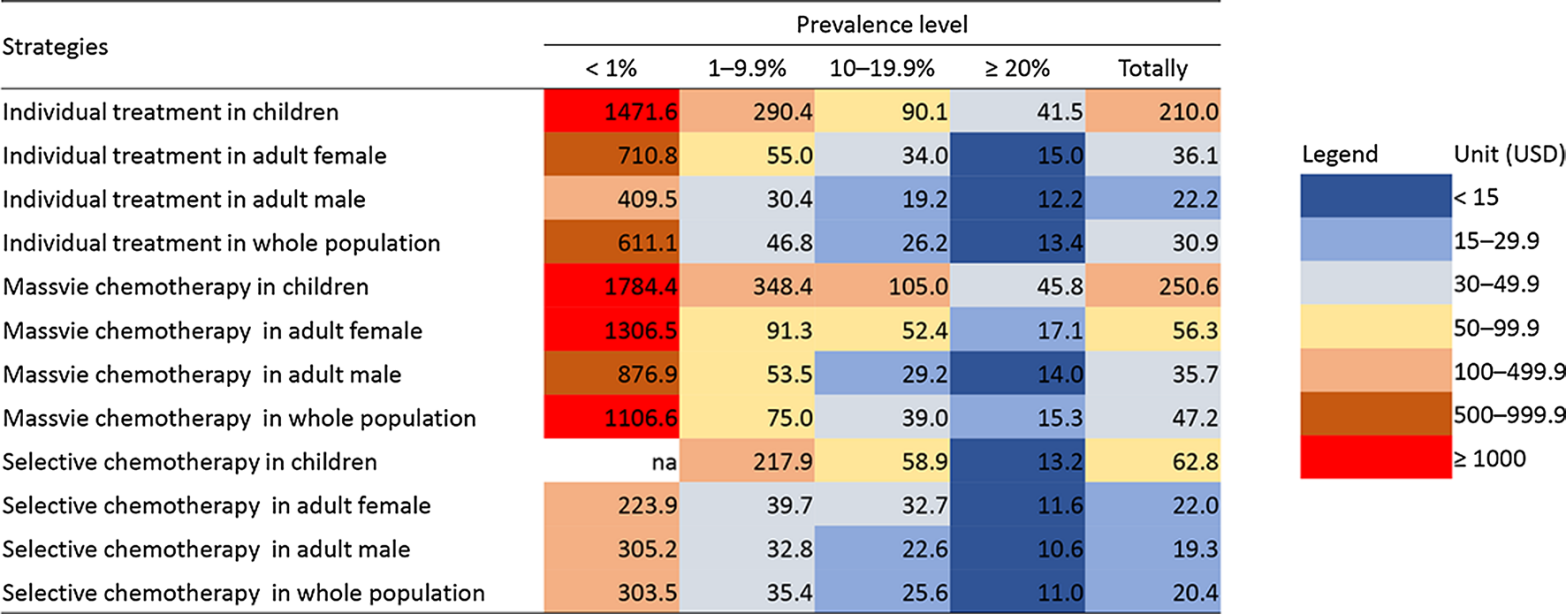
Cost effectiveness of different treatment strategies against *Clonorchis sinensis* infection. *na* not available

In very high prevalence group, cost to treat single infected case in selective chemotherapy was USD 10.6 in adult male, USD 11.6 in adult female, USD 13.2 in children and USD 11.0 overall (Table 1 and Fig. 1). Correspondingly, the cost increased to USD 22.6, USD 32.7, USD 58.9 and USD 25.6 in high prevalence group. In moderate prevalence group, the cost was USD 32.8 in adult male and USD 39.7 in adult female, while it exceeded USD 200.0 in children. In low prevalence group, the cost per infected cases exceeded USD 200.0 in all populations.

**Table 1:** Cost effectiveness (USD) of different treatment strategies against *Clonorchis sinensis* infection

Group	County	Prevalence (%)	Individual treatment in children	Individual treatment in adult female	Individual treatment in adult male	Individual treatment in whole population	Massive chemotherapy in children	Massive chemotherapy in adult female	Massive chemotherapy in adult male	Massive chemotherapy in whole population	Selective chemotherapy in children	Selective chemotherapy in adult female	Selective chemotherapy in adult male	Selective chemotherapy in whole population
Low prevalence	County 1	0.2	na	na	762.1	1555.7	na	na	1642.8	3018.6	na	na	661.4	920.6
	County 2	0.3	na	1162.0	497.1	992.6	na	2142.8	1067.2	1758.6	na	69.3	69.3	89.9
	County 3	0.7	589.7	302.6	546.7	435.4	712.2	550.1	1174.9	746.8	na	na	na	na
	County 4	0.8	na	656.7	217.1	407.6	na	1206.4	459.0	743.8	na	82.3	130.7	122.6
	Subtotal	0.5	1471.6	710.8	409.5	611.1	1784.4	1306.5	876.9	1106.6	na	223.9	305.2	303.5
Moderate prevalence	County 5	5.0	409.9	120.8	39.1	68.0	493.7	213.2	72.4	113.7	44.4	12.2	9.5	10.7
	County 6	7.4	204.9	39.6	25.0	47.4	244.5	62.8	41.8	68.0	na	80.0	42.4	49.1
	County 7	7.9	na	45.0	39.5	45.1	na	72.7	73.3	76.9	na	45.8	47.5	47.3
	County 8	9.8	484.3	54.6	23.8	37.3	584.1	90.6	39.3	59.5	na	26.8	29.8	29.5
	Subtotal	7.5	290.4	55.0	30.4	46.8	348.4	91.3	53.5	75.0	217.9	39.7	32.8	35.4
High prevalence	County 9	13.1	211.5	32.1	24.8	29.4	252.5	48.9	41.3	46.2	33.0	40.2	33.6	36.3
	County 10	15.4	90.3	60.9	14.1	25.7	105.1	102.2	18.2	35.9	na	105.4	12.9	17.6
	County 11	15.5	66.1	24.7	21.7	25.6	75.8	35.1	34.7	37.3	38.5	18.8	22.2	21.5
	County 12	16.5	na	37.3	19.4	24.5	na	58.4	29.7	37.7	na	28.7	23.6	25.0
	Subtotal	15.1	90.1	34.0	19.2	26.2	105.0	52.4	29.2	39.0	58.9	32.7	22.6	25.6
Very high prevalence	County 13	27.2	20.3	18.8	15.9	17.1	20.1	24.2	22.0	22.8	12.1	20.6	18.4	19.1
	County 14	53.7	na	12.7	10.6	11.5	na	12.9	10.5	11.6	na	6.2	7.1	6.8
	Subtotal	40.5	41.5	15.0	12.2	13.4	45.8	17.1	14.0	15.3	13.2	11.6	10.6	11.0
Totally		12.3	210.0	36.1	22.2	30.9	250.6	56.3	35.7	47.2	62.8	22.0	19.3	20.4

*na* not available

In massive chemotherapy, the cost in very high prevalence group was USD 14.0 in adult male, USD 17.1 in adult female, USD 45.8 in children and USD 15.3 overall (Table 1 and Fig. 1). In high prevalence group, the cost was USD 29.2, USD 52.4, USD 105.0 and USD 39.0 respectively. In moderate prevalence group, the cost was USD 53.5 and USD 91.3 in adult male and female, when it exceeded USD 300.0 in children. In low prevalence group, the cost exceeded USD 800.0 in any population.

In individual treatment, the cost in very high prevalence group was USD 12.2 in adult male, USD 15.0 in adult female, USD 41.5 in children and USD 13.4 overall (Table 1 and Fig. 1). The cost doubled nearly in high prevalence group compared to those in very high group. In moderate prevalence group, the cost further increased to USD 30.4 in adult male, USD 55.0 in adult female and USD 290.4 in children and USD 46.8 overall. In low prevalence group, the cost was over USD 400.0 in all populations.

### Cost utility

**Fig. 2:**
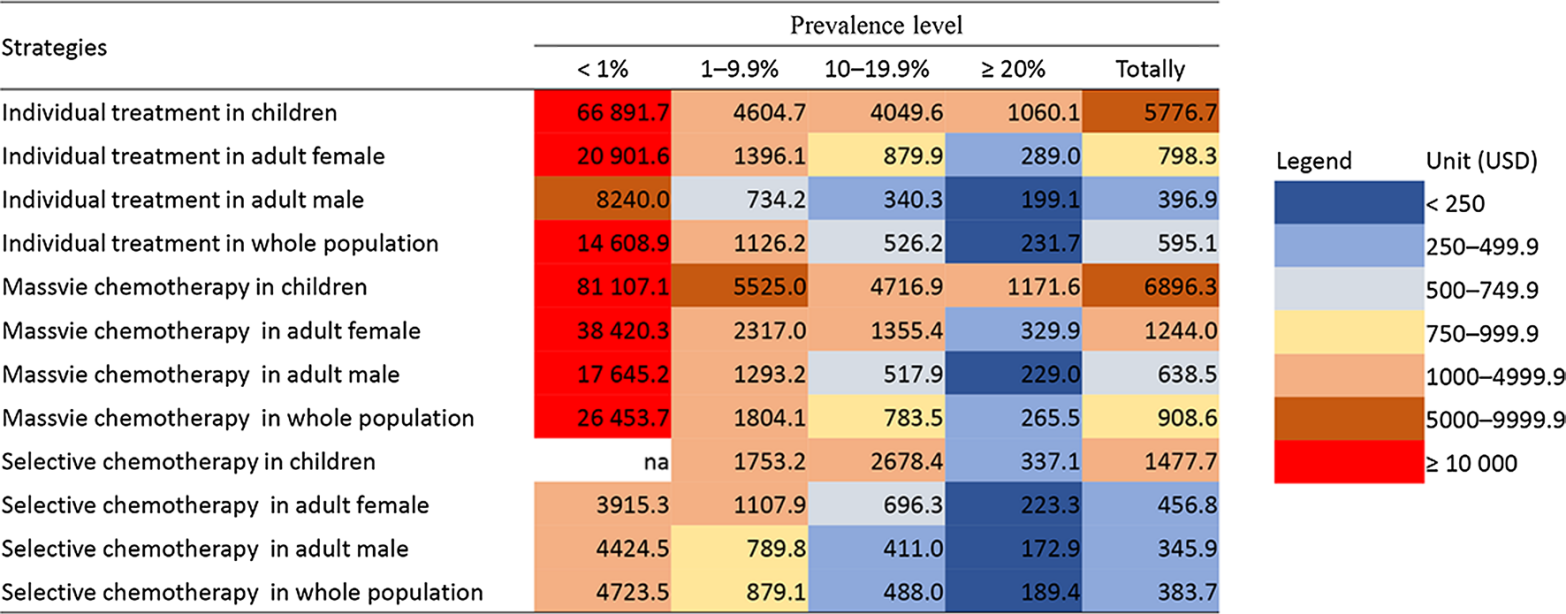
Cost utility of different treatment strategies against *Clonorchis sinensis* infection. *na* not available

In very high prevalence group, cost to avoid per DALYs in selective chemotherapy was USD 172.9 in adult male, USD 223.3 in adult female, USD 337.1 in children and USD 189.4 overall (Table 2 and Fig. 2). Correspondingly, the cost increased to USD 411.0, USD 696.3, USD 2678.4 and USD 488.0 in high prevalence group. In moderate prevalence group, the cost was USD 789.8 in adult male and USD 1107.9 in adult female, while it exceeded USD 1750.0 in children. In low prevalence group, the cost per infected cases exceeded USD 3900.0 in all populations.

**Table 2:** Cost utility (USD) of different treatment strategies against *Clonorchis sinensis* infection

Group	County	Prevalence (%)	Individual treatment in children	Individual treatment in adult female	Individual treatment in adult male	Individual treatment in whole population	Massive chemotherapy in children	Massive chemotherapy in adult female	Massive chemotherapy in adult male	Massive chemotherapy in whole population	Selective chemotherapy in children	Selective chemotherapy in adult female	Selective chemotherapy in adult male	Selective chemotherapy in whole population
Low prevalence	County 1	0.2	na	na	8020.1	16 371.1	na	na	17 287.3	31 764.6	na	na	1 3053.8	1 8170.4
	County 2	0.3	na	13 878.6	7104.9	13 313.6	na	25 591.6	15 252.5	23 588.0	na	827.7	609.9	910.4
	County 3	0.7	26 803.1	12 233.7	20 818.9	17 820.5	32 374.3	22 240.1	44 741.0	30 567.8	na	na	na	na
	County 4	0.8	na	23 717.6	6089.3	12 109.8	na	43 567.3	12 875.2	22 099.0	na	2686.5	3069.4	3349.4
	Subtotal	0.5	6 6891.7	20 901.6	8240.0	14 608.9	81 107.1	38 420.3	17 645.2	26 453.7	na	3915.3	4424.5	4723.5
Moderate prevalence	County 5	5.0	5603.1	4883.8	1487.8	2444.6	6748.6	8619.9	2756.2	4087.4	357.0	493.9	363.7	382.6
	County 6	7.4	3893.0	530.3	437.8	787.5	4645.4	840.3	732.4	1129.3	na	1334.0	760.0	870.8
	County 7	7.9	na	1108.6	866.0	1039.8	na	1792.0	1607.2	1772.9	na	1128.7	1046.3	1094.6
	County 8	9.8	3832.9	2208.5	660.1	1107.1	4623.1	3662.2	1088.1	1767.7	na	1081.6	802.0	873.9
	Subtotal	7.5	4604.7	1396.1	734.2	1126.2	5525.0	2317.0	1293.2	1804.1	1753.2	1107.9	789.8	879.1
High prevalence	County 9	13.1	9612.2	603.2	412.5	517.5	11 476.6	918.0	688.1	813.8	1499.8	628.0	548.4	586.3
	County 10	15.4	4011.4	2461.2	180.3	384.2	4672.8	4130.4	232.3	536.0	na	4261.1	157.2	221.3
	County 11	15.5	3005.1	922.2	559.9	787.4	3444.9	1311.6	894.5	1146.0	1751.0	761.3	671.5	728.2
	County 12	16.5	na	808.7	451.2	559.7	na	1267.5	690.3	860.4	na	622.0	547.4	568.1
	Subtotal	15.1	4049.6	879.9	340.3	526.2	4716.9	1355.4	517.9	783.5	2678.4	696.3	411.0	488.0
Very high prevalence	County 13	27.2	518.8	463.3	321.6	373.9	513.6	596.2	445.7	498.5	310.0	501.9	370.3	413.7
	County 14	53.7	na	217.9	158.3	180.2	na	221.3	157.0	181.0	na	106.8	105.8	106.3
	Subtotal	40.5	1060.1	289.0	199.1	231.7	1171.6	329.9	229.0	265.5	337.1	223.3	172.9	189.4
Totally		12.3	5776.7	798.3	396.9	595.1	6896.3	1244.0	638.5	908.6	1477.7	456.8	345.9	383.7

*na* not available

In massive chemotherapy, the cost in very high prevalence group was USD 229.0 in adult male, USD 329.9 in adult female, USD 1171.6 in children and USD 265.5 overall (Table 2 and Fig. 2). In high prevalence group, the cost was USD 517.9, USD 1355.4, over USD 4700.0 and USD 783.5, correspondingly. In moderate prevalence group, the cost exceeded over USD 1250.0 in all groups, when it was over USD 17 500.0 in any population in low prevalence group.

In individual treatment, the cost in very high prevalence group was USD 199.1 in adult male, USD 289.0 in adult female, USD 1060.1 in children and USD 231.7 overall (Table 2 and Fig. 2). In very high prevalence group, the cost increased to USD 340.3, USD 879.9, USD 4049.6 and USD 526.2, respectively. In moderate prevalence group, the cost further increased to USD 734.2 in adult male and over USD 1000.0 in other populations. In low prevalence group, the cost was over USD 8000.0 in all populations.

### Composition of cost

In individual treatment, the overall composition was 81.6% in diagnosis (fecal examination), 17.4% in purchase of drugs, and another 1.0% in drug delivery (Table 3). The composition of diagnosis was highest in children (98.2%), followed by adult female (84.1%) and then adult male (74.1%). The percentage of diagnosis in overall population reached 57.2% in very high prevalence group, 78.3% in high prevalence group, 87.9% in moderate prevalence group and 99.1% in low prevalence group.

**Table 3:** Cost composition of different treatment strategies against *Clonorchis sinensis* infection

Group	Composition in individual treatment (%)			Composition in massive chemotherapy (%)			Composition in selective chemotherapy (%)		
	Diagnosis	Cost of drugs	Delivery of drugs	Diagnosis	Cost of drugs	Delivery of drugs	Diagnosis	Cost of drugs	Delivery of drugs
Low prevalence	99.1	0.9	0.1	0.0	94.5	5.5	41.9	55.3	2.8
Moderate prevalence	87.9	11.5	0.7	0.0	94.5	5.5	6.7	88.8	4.5
High prevalence	78.3	20.5	1.2	0.0	94.7	5.3	6.4	89.0	4.6
Very high prevalence	57.2	40.5	2.3	0.0	95.0	5.0	3.6	91.7	4.7
Totally	81.6	17.4	1.0	0.0	94.7	5.3	7.5	88.0	4.5

In massive treatment, the overall composition was 94.7% in purchase of drugs and another 5.3% in drug delivery (Table 3). The cost of purchase of drug was 91.8% in children, 94.6% in adult female and 95.4% in adult male. Because all persons in all populations received treatment and the cost in purchase and delivery of drugs was same, thus the composition didn’t vary by prevalence in any single population. However, the cost changed a little in overall population by counties because of the different structure in population and different body weight in different populations.

In selective chemotherapy, the overall composition was 88.0% in purchase of drugs, 7.5% in diagnosis (behavioral screening), and another 4.5% in drug delivery (Table 3). The composition of purchase of drugs was highest in male (90.7%), followed by female (86.3%) and then children (33.0%). The percentage of purchase of drugs in overall population reached 91.7% in very high prevalence group, 89.0% in high prevalence group, 88.8% in moderate prevalence group and 55.3% in low prevalence group.

## Discussion

Adult worms of *C. sinensis* parasitize in human bodies for decades of years [28]. Thus, drug treatment is necessary to control the morbidity and eliminate the infection, which is nowadays also the mainstream intervention against clonorchiasis and other human liver fluke infections [29]. Treatment strategies with high-cost yield are needed in massive control activities [3]. Through large sample, this study demonstrated the cost effectiveness and cost utility of three treatment schedules in different endemic levels and populations. Both cost effectiveness and cost utility were impacted significantly by different treatment strategies, including the endemic levels, targeted populations and treatment schedules.

In this study, both effectiveness and utility indicators were modelled. To our knowledge, no study has yet explored the economic evaluation in term of DALYs in treatment of clonorchiasis. Overall, the evaluation indicated by DALYs is more comprehensive. On one hand, both prevalence and infection intensity are considered in DALYs. Infection intensity indicates the worm burden [30], which is significantly related to the morbidity [11, 31]. On the other hand, not only disability but also death are included in DALYs, which is very important because YLLs could also be caused in clonorchiasis due to cholangiocarcinoma. However, the overall performance in different treatment strategies is similar in both cost effectiveness and cost utility, because high prevalence usually indicates high infection intensity.

The higher the prevalence is, the more cases with *C. sinensis* will be treated, which indicates more cases in share of the huge cost on fecal examination in individual treatment and on drugs in massive and selective chemotherapy. Additionally, in selective chemotherapy, the cost yield was also impacted by the performance of behavioral screening, which is influenced by many factors [17]. In particularly, environmental contamination and subsequently infection in freshwater fish as well as control activities vary by areas [32–34]. However, the screening performance was overall high in those areas with high prevalence (Additional file 1: Table S1).

The different cost yield in different populations was essentially attributable to the difference in prevalence. Because clonorchiasis shows a significantly differential distribution in different genders and ages due to difference in ingesting raw freshwater fish [5, 25, 31]. Adult male shows a higher prevalence compared to adult female and both show higher prevalence compared to children. Thus, in individual treatment and massive chemotherapy, the cost effectiveness in adult male in high endemic level (overall 10–19.9%) was even preferable to that in children in very high endemic level (over 20%), because the prevalence was 23.1% in the former and 8.2% in the latter.

In the same endemic level and same population, cost yield was usually higher in selective chemotherapy compared to individual treatment and massive chemotherapy. Because the cost on fecal examination in individual treatment and drugs in massive chemotherapy was huge, which could be verified by the cost composition, while high screening performance by raw-freshwater fish-eating practice avoided such cost [17]. Especially, the difference between three treatment schedules was very smaller in high endemic level compared to those in low endemic level. When the cost yield in selective chemotherapy declined due to the decreasing performance of screening cases by raw-fish-eating practice in low endemic areas, the cost effectiveness in other two treatment schedules decreased more due to the decline in prevalence. Thus, the difference between selective chemotherapy and another two treatment schedules enlarged when the prevalence became low. However, it was demonstrated the cost yield is not acceptable in any treatment strategy when the prevalence is less than 1%. Thus, new techniques are expected to increase the cost yield in such case [3].

In this study, only monetary cost was considered, when other factors should not be neglected. Individual treatment lies on the definite individual diagnosis. The Kato-Katz method is widely applied because of its simplicity [20, 21]. However, it still takes much time to collect samples, and prepare and examine the smears. For example, it was estimated that the average time to collect a feces sample and perform a single or duplicate Kato-Katz thick smears is about 20 min and 27 min respectively [35]. Obviously, the cost of labor resources is huge. Furthermore, the availability of enough technicians in large field surveys is also challenging. Additionally, it should also be considered that in low endemic situations (low prevalent areas and populations), the diagnostic sensitivity of Kato-Katz method decreases [36]. On the comparison, the time spent in screening whole population for treatment in selective chemotherapy and delivery of drugs to whole population in massive chemotherapy is significantly less. On the other hand, all infected cases could be treated in both individual treatment and massive chemotherapy regardless of the incompliance and the possible low sensitivity of fecal examination in low prevalence, while it is hard to cover all infected cases in selective chemotherapy because the sensitivity of behavioral screening is usually less than 100%. However, a higher cost yield in selective chemotherapy indicates more cases to be treated in given resource.

It must be noticed this study advocates the distribution of treatment resources to prioritized areas and populations, which doesn’t indicate the unimportance of low endemic areas and low prevalent population (i.e., children). New techniques should be developed to detect the cases in these areas and populations with low prevalence. The drug-taking compliance probably varies in different treatment schedules, endemic levels and populations, which was not considered in this study and deserves to be explored in future. Additionally, drug efficacy may also vary in different endemic levels and populations due to difference in infection intensity, which also needs to be explored.

This study had several limitations. First, the indicator of cost effectiveness and utility are both deterministic without confidential interval, because the prevalence, diagnostic performance of fecal examination and behavioral screening as well as the cost were all deterministic in this study. In future studies, the uncertainty in diagnosis and the difference on cost by areas should be considered. Second, only short-term effectiveness and utility were modelled. The screening performance of behavior and the compliance of drug-taking after multi-round treatment, and other factors had not be considered. Transmission dynamic model is expected to further illuminate them in future.

## Conclusions

This study demonstrates a significant variation of cost yield by different treatment strategies including three treatment schedules, four endemic levels, and three types of populations. Although cost yield in high endemic areas (over 10% in prevalence) is approaching, chemotherapy is more acceptable because of the huge labor input in diagnosis in individual treatment. Additionally, selective chemotherapy demonstrates a little higher yield compared to massive chemotherapy. Relatively, the cost yield is higher in adults especially men compared to children. In moderate endemic areas (1–9.9% in prevalence), the cost yield in different treatment schedules all decreases, but selective chemotherapy targeting adults may still be considered. However, in low endemic areas (< 1% in prevalence), although selective chemotherapy shows higher cost yield compared to other two schedules, the cost is too high to be acceptable in any strategy. Thus, new techniques should be explored. Overall, to be cost effective, high endemic areas and adults especially men could be prioritized, and chemotherapy especially the selective one is of first choice.

## Supplementary Information

**Additional file 1: Table S1.** Epidemiological profiles of *Clonorchis sinensis* infection and raw-freshwater fish-eating practice. **Table S2.** Disability-adjusted life years caused by *Clonorchis sinensis* infection by counties, endemic levels and populations.

## Data Availability

All data supporting the findings of this study are included in the article and additional file.

## Abbreviations

DALYs: Disability-adjusted life years
YLDs: Life living with a disability
YLLs: Years of life lost
EPG: Eggs per gram of feces
GMEPG: Geometric mean of EPG
