# Supplementary material for: Cost yield of different treatment strategies against Clonorchis sinensis infection
Source: Infect Dis Poverty. 2021 Dec 22;10:136. doi: 10.1186/s40249-021-00917-1 (PMC8693485; doi:10.1186/s40249-021-00917-1)
Supplement: Supplementary file 1 — Additional file 1: Table S1. Epidemiological profiles of Clonorchis sinensis infection and raw-freshwater fish-eating practice. Table S2. Disability-adjusted life years caused by Clonorchis sinensis infection by counties, endemic levels and populations. [file 40249_2021_917_MOESM1_ESM.docx]

**Table S1: Epidemiological profiles of *Clonorchis sinensis* infection and raw-freshwater fish-eating practice^*^**

| Group | County | Not eating raw freshwater fish | | | Eating raw freshwater fish | | | Totally | | | Screening sensitivity of raw-fish-eating practice (%) |
| --- | --- | --- | --- | --- | --- | --- | --- | --- | --- | --- | --- |
|  |  | No. participants | Infection | Prevalence (%) | No. participants | Infection | Prevalence (%) | No. participants | Infection | Prevalence (%) |  |
| Low | County 1 | 881 | 1 | 0.1 | 119 | 1 | 0.8 | 1000 | 2 | 0.2 | 50.0 |
|  | County 2 | 950 | 1 | 0.1 | 5 | 2 | 40.0 | 955 | 3 | 0.3 | 66.7 |
|  | County 3 | 1110 | 8 | 0.7 | 0 | 0 | na | 1110 | 8 | 0.7 | 0.0 |
|  | County 4 | 1024 | 6 | 0.6 | 13 | 2 | 15.4 | 1037 | 8 | 0.8 | 25.0 |
|  | Subtotally | 3965 | 16 | 0.4 | 137 | 5 | 3.6 | 4102 | 21 | 0.5 | 23.8 |
| Moderate | County 5 | 973 | 4 | 0.4 | 53 | 47 | 88.7 | 1026 | 51 | 5.0 | 92.2 |
|  | County 6 | 592 | 17 | 2.9 | 408 | 57 | 14.0 | 1000 | 74 | 7.4 | 77.0 |
|  | County 7 | 458 | 4 | 0.9 | 545 | 75 | 13.8 | 1003 | 79 | 7.9 | 94.9 |
|  | County 8 | 664 | 14 | 2.1 | 384 | 89 | 23.2 | 1048 | 103 | 9.8 | 86.4 |
|  | Subtotally | 2687 | 39 | 1.5 | 1390 | 268 | 19.3 | 4077 | 307 | 7.5 | 87.3 |
| High | County 9 | 516 | 44 | 8.5 | 484 | 87 | 18.0 | 1000 | 131 | 13.1 | 66.4 |
|  | County 10 | 853 | 86 | 10.1 | 173 | 72 | 41.6 | 1026 | 158 | 15.4 | 45.6 |
|  | County 11 | 825 | 97 | 11.8 | 180 | 59 | 32.8 | 1005 | 156 | 15.5 | 37.8 |
|  | County 12 | 386 | 1 | 0.3 | 614 | 164 | 26.7 | 1000 | 165 | 16.5 | 99.4 |
|  | Subtotally | 2580 | 228 | 8.8 | 1451 | 382 | 26.3 | 4031 | 610 | 15.1 | 62.6 |
| Very high | County 13 | 209 | 6 | 2.9 | 791 | 266 | 33.6 | 1000 | 272 | 27.2 | 97.8 |
|  | County 14 | 474 | 20 | 4.2 | 526 | 517 | 98.3 | 1000 | 537 | 53.7 | 96.3 |
|  | Subtotal | 683 | 26 | 3.8 | 1317 | 783 | 59.5 | 2000 | 809 | 40.5 | 96.8 |
| Totally |  | 9915 | 309 | 3.1 | 4295 | 1438 | 33.5 | 14210 | 1747 | 12.3 | 82.3 |

^*^ Data in this table had already been demonstrated partially elsewhere, which was used for overall cost effectiveness analysis and thus demonstrated here again.

Reference: Qian MB, Jiang ZH, Ge T, Wang X, Zhou CH, Zhu HH, et al. Rapid screening of *Clonorchis sinensis* infection: Performance of a method based on raw-freshwater fish-eating practice. Acta Trop. 2020;207:105380. na: not available.

**Table S2: Disability-adjusted life years caused by *Clonorchis sinensis* infection by counties, endemic levels and populations**

| Group | County | Prevalence (%) | Children | | | Adult female | | | Adult male | | | Totally | | |
| --- | --- | --- | --- | --- | --- | --- | --- | --- | --- | --- | --- | --- | --- | --- |
|  |  |  | YLDs/1000 | YLLs/1000 | DALYs/1000 | YLDs/1000 | YLLs/1000 | DALYs/1000 | YLDs/1000 | YLLs/1000 | DALYs/1000 | YLDs/1000 | YLLs/1000 | DALYs/1000 |
| Low | County 1 | 0.2 | 0.0 | 0.0 | 0.0 | 0.0 | 0.0 | 0.0 | 0.4 | 0.0 | 0.4 | 0.2 | 0.0 | 0.2 |
|  | County 2 | 0.3 | 0.0 | 0.0 | 0.0 | 0.2 | 0.0 | 0.2 | 0.4 | 0.0 | 0.4 | 0.2 | 0.0 | 0.2 |
|  | County 3 | 0.7 | 0.1 | 0.0 | 0.1 | 0.2 | 0.0 | 0.3 | 0.1 | 0.0 | 0.2 | 0.2 | 0.0 | 0.2 |
|  | County 4 | 0.8 | 0.0 | 0.0 | 0.0 | 0.1 | 0.0 | 0.1 | 0.5 | 0.1 | 0.5 | 0.2 | 0.0 | 0.3 |
|  | Subtotal | 0.5 | 0.0 | 0.0 | 0.0 | 0.1 | 0.0 | 0.1 | 0.3 | 0.0 | 0.4 | 0.2 | 0.0 | 0.2 |
| Moderate | County 5 | 5.0 | 0.6 | 0.0 | 0.6 | 0.6 | 0.1 | 0.7 | 2.0 | 0.4 | 2.4 | 1.2 | 0.2 | 1.4 |
|  | County 6 | 7.4 | 0.8 | 0.0 | 0.8 | 6.6 | 0.2 | 6.8 | 8.5 | 0.7 | 9.2 | 4.2 | 0.3 | 4.5 |
|  | County 7 | 7.9 | 0.0 | 0.0 | 0.0 | 3.0 | 0.2 | 3.2 | 3.8 | 0.4 | 4.2 | 3.1 | 0.3 | 3.4 |
|  | County 8 | 9.8 | 0.8 | 0.0 | 0.8 | 1.4 | 0.2 | 1.6 | 5.5 | 0.7 | 6.2 | 2.9 | 0.4 | 3.3 |
|  | Subtotal | 7.5 | 0.7 | 0.0 | 0.7 | 2.3 | 0.2 | 2.5 | 4.7 | 0.5 | 5.2 | 2.9 | 0.3 | 3.1 |
| High | County 9 | 13.1 | 0.3 | 0.0 | 0.3 | 5.9 | 0.3 | 6.3 | 9.1 | 0.7 | 9.8 | 7.0 | 0.5 | 7.4 |
|  | County 10 | 15.4 | 0.8 | 0.0 | 0.8 | 1.2 | 0.2 | 1.4 | 27.4 | 1.6 | 29.0 | 9.7 | 0.6 | 10.3 |
|  | County 11 | 15.5 | 1.1 | 0.0 | 1.1 | 3.9 | 0.4 | 4.4 | 6.7 | 0.8 | 7.5 | 4.5 | 0.5 | 5.0 |
|  | County 12 | 16.5 | 0.0 | 0.0 | 0.0 | 4.3 | 0.3 | 4.5 | 8.8 | 1.0 | 9.8 | 6.6 | 0.6 | 7.2 |
|  | Subtotal | 15.1 | 0.8 | 0.0 | 0.8 | 3.9 | 0.3 | 4.2 | 12.0 | 1.0 | 13.0 | 7.0 | 0.6 | 7.5 |
| Very high | County 13 | 27.2 | 7.3 | 0.0 | 7.3 | 9.0 | 0.6 | 9.6 | 13.8 | 1.3 | 15.1 | 11.5 | 1.0 | 12.4 |
|  | County 14 | 53.7 | 0.0 | 0.0 | 0.0 | 24.7 | 1.2 | 26.0 | 40.2 | 2.7 | 42.9 | 32.3 | 2.0 | 34.3 |
|  | Subtotal | 40.5 | 3.2 | 0.0 | 3.2 | 16.5 | 0.9 | 17.4 | 27.4 | 2.0 | 29.4 | 21.9 | 1.5 | 23.4 |
| Totally | | 12.3 | 0.5 | 0.0 | 0.5 | 4.3 | 0.3 | 4.6 | 9.7 | 0.8 | 10.5 | 5.9 | 0.5 | 6.4 |

YLDs: Years of life living with a disability; YLLs: Years of life lost; DALYs: Disability-adjusted life years.
